# Effect of Bi and Ca on the Solidification Parameters of Sr-Modified Al-S-Cu (Mg) Alloys

**DOI:** 10.3390/ma15196903

**Published:** 2022-10-05

**Authors:** Shimaa El-Hadad, Agnes M. Samuel, Fawzy H. Samuel, Herbert W. Doty, Victor Songmene

**Affiliations:** 1 Department of Casting Technology, Central Metallurgical Research and Development Institute, Cairo 11421, Egypt; 2 Dept. des Sciences appliquées, Université du Québec à Chicoutimi, Chicoutimi, QC G7H 2B1, Canada; 3 Materials Technology, General Motors Global Technology Center, Warren, MI 48093-2350, USA; 4 École de Technologie Supérieure, Université du Québec, Montréal, QC H3C 1K3, Canada

**Keywords:** tramp elements, thermal analysis, solidification rate, interface velocity, thermal gradient

## Abstract

The effect of tramp elements, mainly Bi and Ca, on the thermal characteristics of Sr-modified Al–Si–Cu and Al–Si–Cu–Mg alloys has been investigated using thermal analysis, X-ray radiography, and field emission scanning electron microscopy (FESEM) techniques. The high affinity of Bi to interact with Sr results in an increase in the Al-Si eutectic temperature, and hence an increase in the size of eutectic silicon particles. In contrast, the Ca–Sr interaction seems to have no significant effect on the alloy thermal behavior. The effect of these interactions on porosity formation has been discussed. Hot zones may be formed in thin cavities, in particular, near the bottom of the mold, leading to formation of unexpected coarse porosity, mostly shrinkage type. The study also highlights the significance of other parameters on porosity formation, such as no melt degassing, SrO, Al_2_O_3_ (strings or bifilms), as well as the presence of iron-based intermetallics.

## 1. Introduction

Studying the effect of Bi and Ca additions on the structure and properties of Al–Si-based cast alloys was the objective of several studies done by the present research group and other researchers. Bismuth levels of up to 0.5% may be tolerated in wrought alloys to improve machinability: The presence of Bi is known to increase the machining speed and reduce the need for cutting fluids [1]. The addition of Bi to alloy 356.2 (containing Al, 7%Si, 0.36%Mg) also increases the level of microshrinkage [2,3], whereas Ca refines and spheroidizes the iron intermetallics, as well as the eutectic silicon in Al–Si-based alloys, resulting in improved mechanical properties. In addition, Ca increases the hydrogen solubility in the aluminum melt at trace concentration levels and is often responsible for casting porosity.

Another study reported on the effect of Ca–Sr–Mg and Bi–Sr–Mg interactions on the microstructural characterization and tensile properties of B319 Alloy (Al, 6.6%Si, 3.7%Cu, 0.4%Mg) [4]. The presence of calcium as impurity is usually attributed to low-purity commercial silicon used in the production of Al–Si alloys. The recommended maximum amount of calcium tolerable in aluminum alloys varies according to individual research. It was reported that calcium gives rise to porosity, micro-cavitation, as well as alloy fluidity. Hence, an amount of Ca content of about 0.003% (30 ppm) would be a tolerable amount in Al–Si alloys, since it has deleterious effects on fluidity and shrinkage properties [5].

The results of Farahany et al. [6] on the effect of Bi, Sr, and Sb on the solidification behavior of A383 die casting alloy (Al, 2.5%Cu, 1.3%Fe, 3%Zn, 6.5%Si, 0.5%Mn) within solidification rates of 0.6 °C/s –2 °C/s showed that both Bi and Sb caused an increase in recalescence with increased solidification rate, whereas Sr addition reduced the recalescence of the Al-Si eutectic reaction. The Al-Si eutectic solid fraction (liquid to solid) increased in the order of Sr > Bi > Sb. In another study by Farahany et al. [7] on an Al–Si–Cu–Zn alloy with addition of Bi, the authors reported that during solidification, Bi is pushed to the solid–liquid interface. High concentration of Bi would reduce the surface tension of aluminum surrounding the Si particles. As a result, the nucleation temperature T_N_ and growth temperature T_G_ of Al-Si phase reduced with addition of Bi. The undercooling temperature (T_N__T_Min_) and time did not change and remained stable, and the growth temperature T_G_ of Al-Si eutectic decreased with the increase in the added Bi.

The effects of Cu, Co, Ni, Sb and Bi and growth rates on the microstructure of the directionally solidified Al–12.6 wt.%Si–X alloys have been investigated by Kaya and Aker [3]. These alloys were directionally solidified under a constant temperature gradient and different growth rates (8.3–166.0 μm/s). According to experimental results, the microstructure of the solidified Al-Si-X samples changes with alloying elements (Cu, Co, Ni, Sb, and Bi) and the growth rate. With respect to Bi and Ca elements, the presence of Bi is known to increase the machining speed and reduce the need for cutting fluids, whereas Ca refines and spheroidizes the iron intermetallics, as well as the eutectic silicon in Al–Si-based alloys, resulting in improved mechanical properties [4,8,9].

The characteristic temperatures, including nucleation and growth temperatures of eutectic Al–Si alloys, were analyzed [10,11]. Bismuth additions (0–2 wt.%) to Al-17.5%Si alloy resulted in modifying and refining the Si particles into small pentagonal-shaped particles with blunt corners. Volume fraction, average equivalent diameter, and aspect ratio of the Si_P_ particles decreased with increase in Bi concentration in the alloy [12]. The solubilities of Al and Sr in Si after solidification were determined to be 430 ± 160 at-ppm and 40 ± 10 at-ppm, respectively. Strontium predominantly segregates to the Si phase confirming its importance in the modification of the eutectic growth [9,13]. Strontium oxide (SrO) forms during melting of Sr-modified alloys. It is inevitable and cannot be removed completely regardless of the filtration system used during pouring. The crystal structure of SrO is a cubic structure, belonging to the cF8 group (∆H = −592.0 kJ·mol^−1^, exothermic, ∆G = −560.6 kJ/mol, at 0.1 MPa) [14].

Thermodynamically, when its overall Gibbs-free energy changes, ∆G_f_ is negative. For reaction [1], the Gibbs-free energy change under isobaric conditions is given as:

∆G = ∆G° + RT ln K_1_ (1)

where ∆G is the standard Gibbs-free energy of the formation of the oxide at absolute temperature, T and R is the gas constant. If ∆G = 0, the system is at equilibrium, and if ∆G > 0, the reaction is thermodynamically unfavorable. At equilibrium (∆G = 0),

∆G = −R T Ln K1= R T ln P_O2_ (2)

where

K1 = 1/P_O2_ (3)

The oxygen partial pressure, P_O2_, can be read directly from the Ellingham diagram [15] by using the P_O2_ scale along the bottom and the right side of the diagram. A straight line drawn from the index point labeled ‘O’ (at ∆G = 0, T = 0 K) at the upper left of the diagram, through a specific temperature point on an oxide line, intersects the P_O2_ scale at the dissociation oxygen partial pressure (P^diss^) for that oxide at that particular temperature. Accordingly, the oxides lower on the diagram are more stable and, consequently, have lower P^diss^ values [16,17,18].

In a previous study, the authors’ group highlighted the effect of Ca–Sr–Mg and Bi–Sr–Mg interactions on the microstructure and tensile properties of Sr-modified Al–Si–Cu (Mg) alloys [4]. The present investigation was undertaken to emphasize the role of Bi and Ca on the solidification thermal parameters of the Al–Si–Cu (Mg) alloys, such as the undercooling, the thermal gradient, and the solid/liquid interface velocity, and hence on the solidified microstructure, in particular, porosity formation. In addition, the present study also aims at demonstrating how the additions of Bi and Ca are related to the observed cast structures of these alloys. These elements are designated “tramp elements” because they are not included in the original alloy chemical specifications.

## 2. Experimental Procedures

**Figure 1:**
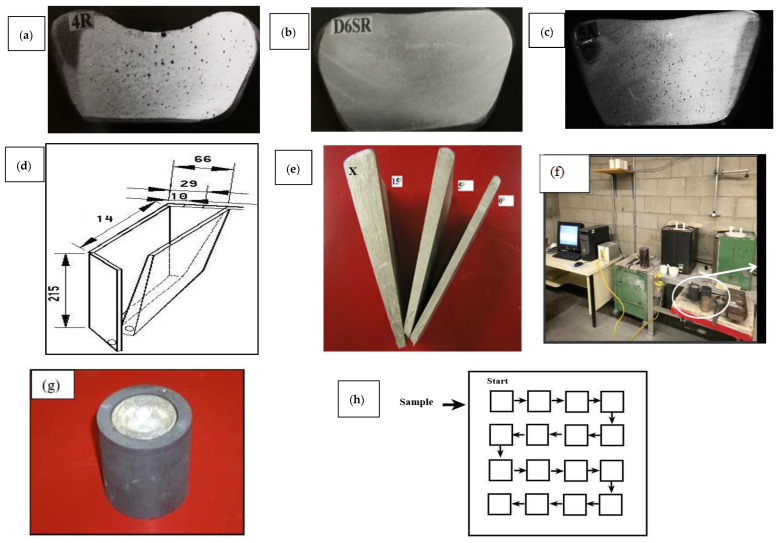
Examples of RPT test samples obtained from Al–Si–Cu (Mg) alloy melts: (**a**) before degassing, (**b**) 0.1 mL/100 g Al, (**c**) 0.3 mL/100 g Al. (**d**) Schematic diagram of the variable angle wedge mold and the position of used thermocouples; (**e**) Variable angle mold castings. All dimensions are in mm. (**f**) Slow cooling rate casting set-up, with smaller electrical resistance furnace, and (**g**) cylindrical graphite mold used for casting. (**h**) Schematic diagram showing the sequence of measuring porosity—each square represents one field.

The alloys were melted in a 7 kg capacity silicon carbide crucible, using an electrical resistance furnace. The melting temperature was kept at 750° ± 5 °C. The melt was degassed with argon for twenty minutes using a rotary impeller degassing system (150 rpm speed). The melts were modified using strontium (~150 ppm). Strontium was added in the form of Al-10%Sr master alloy. Trace additions of Bi and Ca were made using Al-5% Bi and Al-10% Ca master alloys. Table 1 lists the chemical compositions of the A319.2 (coded B) and B319.2 (coded C) alloys that were used to prepare the various Bi- and Ca-containing alloys listed in Table 2. The tolerable concentrations of Ca and Bi in Al–Si–Cu (Mg) cast alloys are 0.002–0.004% (20–40 ppm) and Sr/Bi > 0.45, respectively [19]. The hydrogen content in the molten bath was monitored using both an AlScan apparatus, as well as reduced pressure tests (RPT), as shown in Figure 1a–c.

It should be pointed out that, although the concentrations of Bi and Ca used in the present study are higher than those normally obtained in piston type alloys, these high concentrations were employed for the purposes of assessing their influence on the solidification of Sr-modified Al–Si–Cu alloys. In addition, compared to trace additions, high concentrations are more controllable and reproducible in laboratory experiments.

The melts were poured into a variable angle wedge mold manufactured from mild steel (see Figure 1d). The inside of the mold was spray-coated with a thin layer of vermiculite, about 60µm. The mold was variously adjusted to angles of 0, 5, and 15 degrees in order to incorporate the effect of different solidification rates. The casting molds/castings corresponding to these three angles were termed small, medium, and large, respectively. Thermocouples were positioned along the vertical centerline of the casting using holes drilled through the face of the mold. In our experiments, the thermocouples were located at 2.5, 4, 6.5, 7.5, 11.5, and 17.5 cm from the bottom of the mold—Figure 1e. The size of the drilled holes to place the thermocouple was about 2 mm. The temperature-time data were collected using a high-speed data acquisition system linked to a computer system to record the temperature-time data every 0.1 s. Temperature gradients and interface velocities were measured only in the vertical direction, although the wedge mold also exhibited temperature gradients in the horizontal direction.

Due to the lack of directional solidification in the latter case, the horizontal interface velocity was not measured. It should be emphasized here that the interface velocity refers to that in the vertical direction. Before pouring, the mold was inclined at 35 degrees with respect to the vertical position, and then slowly tilted up during pouring to minimize turbulence effects. In some cases, traditional thermal analysis was carried out on the B and C alloys (details are described elsewhere [19]). The solidification rate (based on dendrite arm spacing) was calculated to be in the range 0.3–0.8 °C/s.

Three pairs of thermocouples (chromel-alumel, type K) were each placed at positions corresponding to those from which samples from the casting were later sectioned for metallographic examination. The temperature-time data were obtained using a high-speed data acquisition system linked to a computer (at a rate of 0.02/s). Samples for chemical analysis were taken simultaneously for each melt condition. For metallographic examination, three samples were sectioned from each mold casting, corresponding to the three positions at which the thermocouple pairs were used to record the thermal data. The surface containing the thermocouple tip was polished in each case (1 µm diamond paste). Samples were also sectioned from the casting for radiographic examination.

Thermal analysis was carried out in order to follow on the sequence of phases precipitated during solidification, using a smaller electrical resistance furnace with a cylindrical graphite crucible of 2 kg capacity. The melting temperature was also maintained at 800 °C. The molten metal was poured into an 800 gm capacity graphite mold preheated to 650 °C to obtain near equilibrium solidification conditions (0.35 °C s^−1^). The arrangement used for producing the slow cooling rate castings is shown in Figure 1f,g.

For selected alloys/conditions, samples (2 cm × 2 cm) were sectioned from the casting centerline at different opening angles for preparing metallographic samples for porosity measurements. Each sample was mounted individually in bakelite, and then ground and polished to obtain a mirror-like surface. The samples were examined using an Olympus PMG3 optical microscope connected to a Clemex image-analysis system. In each case, an average of 50 readings at 100X was reported, as depicted in Figure 1h.

A Hitachi-SU8000 field-emission scanning electron microscope (FESEM), as was used in this study, can provide clear and less electrostatically distorted high-resolution images even at low voltages, with an image resolution of 2.1 nm at 1 kV, and 1.5 nm at 15 kV. The FESEM instrument also comes equipped with a standard secondary electron detector (SE), a backscatter electron detector (BSE), an energy dispersive X-ray spectrometer (EDS), and a wavelength dispersive X-ray spectrometer (WDS).

## 3. Results and Discussion

### 3.1. Porosity Formation

**Figure 2:**
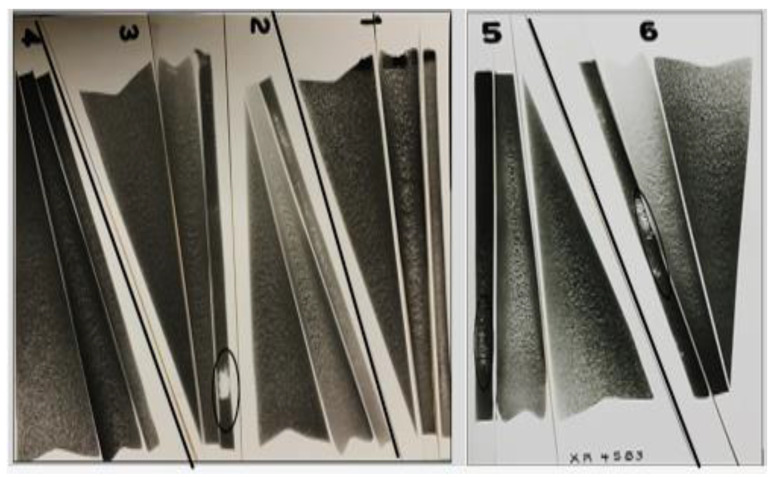
X-ray radiographs taken from several castings at the three opening angles shown in Figure 1e. The numbers 1 to 6 simply represent a batch of castings taken from a specific melt in each case.

Figure 2 shows a series of X-ray radiographs for samples sectioned from various castings obtained at the three angles. In all cases, a hot zone was observed in the small angle mold casting, as a result of the solidification of liquid metal in contact with the mold walls while the metal in the center was still in the molten state, forming pockets of porosity, as illustrated by the circled areas. The variation in severity of these pockets depends on both the temperature of the mold and that of the liquid metal. It is evident from Figure 2 that conditions 1, 2 and 4 are the best casting conditions (i.e., with respect to the mold and molten metal temperatures) to avoid occurrence of hot zones.

Table 3a,b depicts the porosity characteristics obtained from the studied alloys (small and large angles) when the mold was heated at about 325 °C. It is inferred from Table 3a,b that measuring porosity from areas away from the hot zones reveals very low porosity size, mostly due to SrO formed during pouring of the molten metal or during holding.

**Figure 3:**
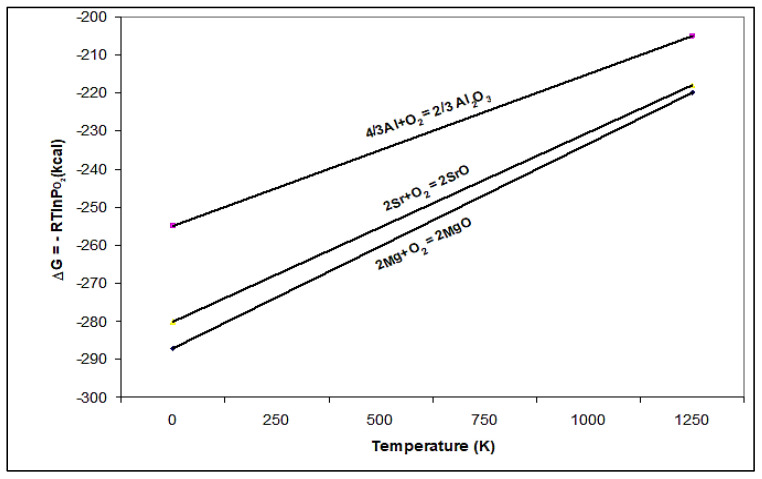
Calculated free energy for Sr oxidation.

**Figure 4:**
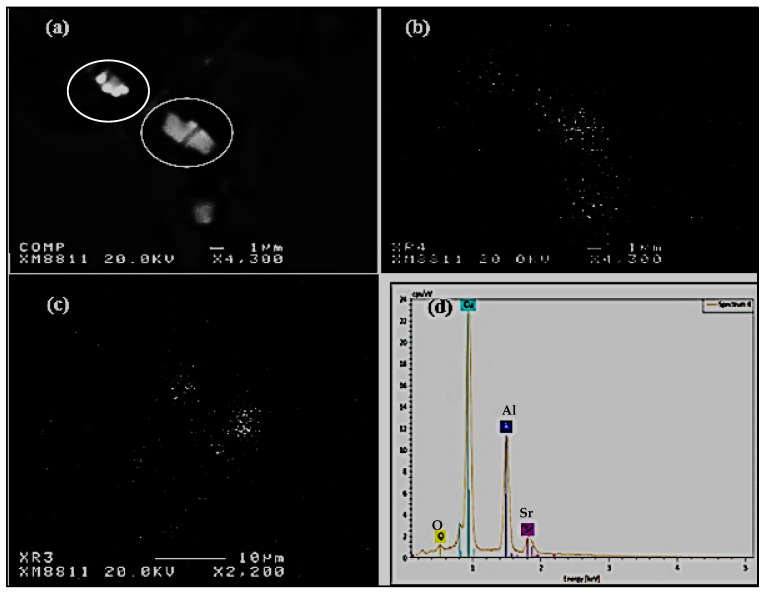
An example of SrO particles observed in the large section-marked X in Figure 1e: (**a**) backscattered electron image, (**b**,**c**) Sr and O distribution in (**a**), respectively, (**d**) EDS spectrum corresponding to circled particle in (**a**) revealing peaks due to Sr and O.

From the Ellingham diagram [15] and Figure 3 (our calculation) it can be seen that Ca and Mg are among the metals that form stable oxides, whereas Sr falls between Mg and Al. No thermodynamic data are available for Bi. However, the work of El-Hadad et al. [19] reveals that Bi has a high affinity for oxidation. Thus, combining all this information would explain the data reported in Table 3. Obviously, increasing the wedge angle to 15° would increase the solidification time, and hence the resulting porosity. Figure 4 shows an example of the SrO particles observed in the present study.

**Figure 5:**
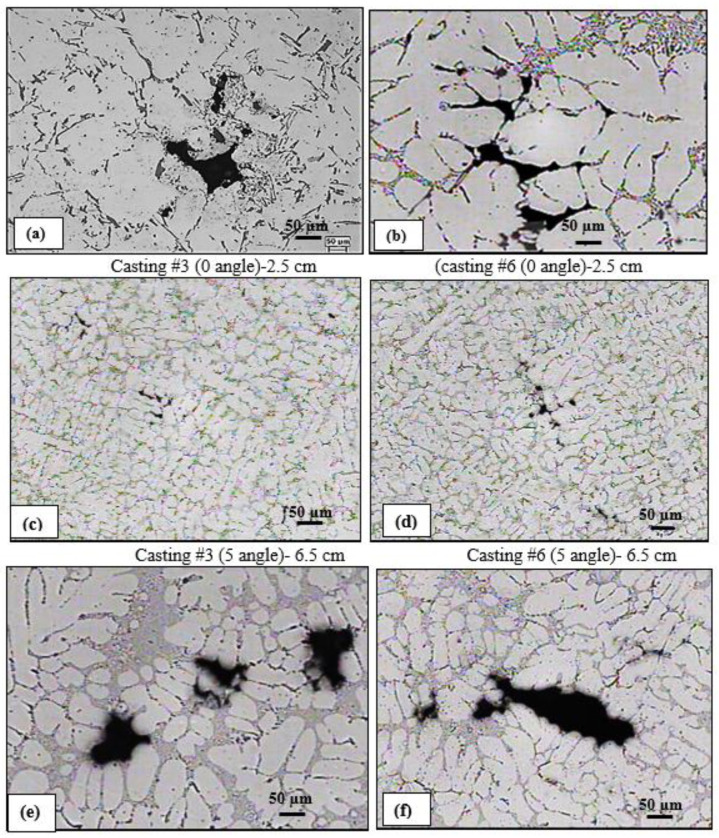
(**a**–**f**) Effect of solidification rate (measured as a function of distance from the mold bottom) on porosity formation in base alloys. Casting #3 (B alloy), casting #6 (C alloy).

Figure 5a,b show examples of porosity viewed from the hot zones revealing severe shrinkage cavities caused by solidification of liquid metal surrounded by solidified metal, as was pointed out in Figure 1. Samples prepared from the same alloys, at 5° opening angle (medium), and 6.5 cm away from the bottom of the mold reveal the presence of fine shrinkage or a mixture of gas and shrinkage porosity scattered throughout the sample, whereas those taken from the same casting at 11.5 cm away from the bottom of the mold (15 deg angle) show fairly large porosity, similar to those obtained in Figure 5a,b, mainly the shrinkage type (Figure 5e,f) due to the increase in the solidification time.

**Figure 6:**
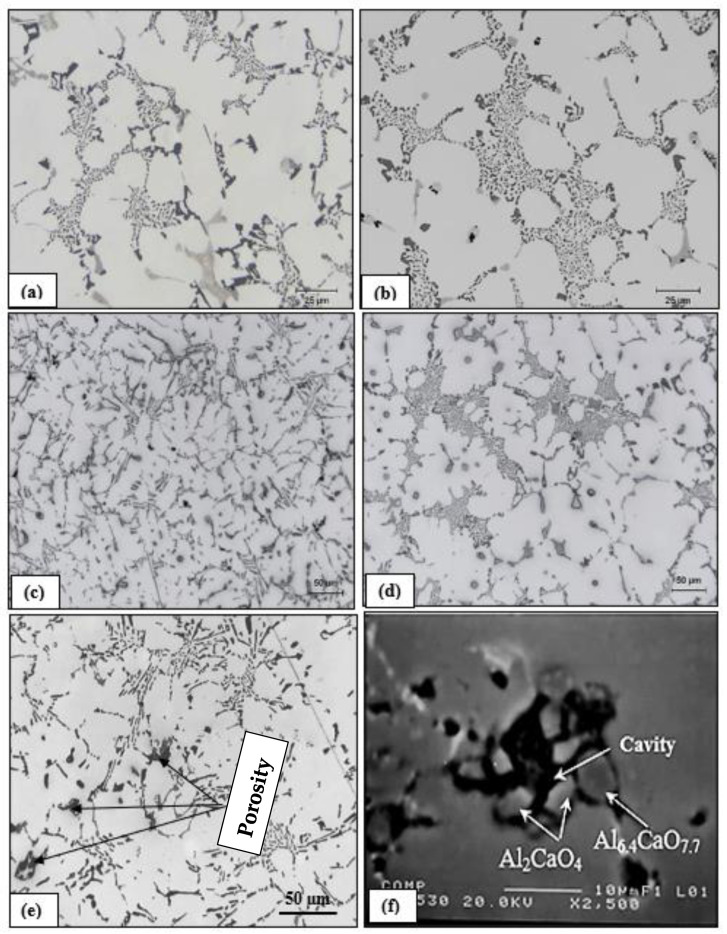
(**a**,**c**,**e**) B, BB2 and BB6 alloys, respectively, (**b**,**d**,**f**) C, CC and CC5 alloys, respectively (medium mold, mold angle 5°). Note the de-modification effect of Bi, compared to that of Ca. In all cases, thermocouples were placed ~6.5 cm from the bottom, away from the shrinkage cavity). Gray compounds in (**f**) are mostly Al-Ca-O oxides.

Figure 6 demonstrates the effect of addition of Bi and Ca on porosity formation in alloys B and C. Another observation to be considered is the affinity of Bi to react with Sr leading to a clear reduction in its modification effect, compared to the influence of Ca addition. In most cases, Bi and Ca showed a clear tendency to react with oxygen leading to the formation of oxides.

Solidus velocity is one of the strongest factors controlling hydrogen porosity formation, since pore formation is a diffusion-controlled process. Thus, a slow-moving interface (i.e., slow solidification rate) will allow diffusion of hydrogen, leading to pore growth. Therefore, the role of Bi and Ca in lowering the solidus velocity is to promote the formation and growth of pores [20]. Another parameter to consider is the role of the crystal lattice in determining the alloy strength. In this context, Nguyen and Bonamy [21] investigated dependence of a material’s resistance to failure on the atomistic scale. Their results lead to the conclusion that fracture toughness depending on the lattice geometry is incompatible with Griffith’s relationship between fracture and free surface energy.

According to Battle and Pehlke [22], the partition coefficient (*k*_0_) is defined as:

*k*_0_ = *C*_*s*_/*C*_*L*_ (4)

where *C_s_* and *C_L_* are the concentrations of solute atoms in solid and liquid at the interface, respectively. The Scheil equation is defined as:

(5) $$C_{S}^{i}=C_{0}k_{0}{\left(1-f\right)}^{-\left(1-k_{0}\right)}$$

where *C*_0_ and *f* are the nominal composition and the solid mass fraction of alloy, respectively. Valdes et al. [23] proposed the following equation to calculate *k_0_* during solidification:

(6) $$k_{0}^{Exp-2}=\frac{C_{0}-C_{L}f_{L}}{1-f_{L}}$$

From thermal analysis parameters (obtained at 0.8 °C/s) coupled with electron probe microanalysis (EPMA) results, and taking into consideration the large number of phases in the present alloy, the partition coefficient values of Bi and Ca are approximately 0.19 and 0.32, respectively.

Fuller and Ditzenberger [24] expressed the diffusivity of bismuth in the 1220–1380 °C temperature range as D = 1030 × e^(−1.109eV/kT)^ cm^2^/s, with an estimated error of about ±40%.

**Figure 7:**
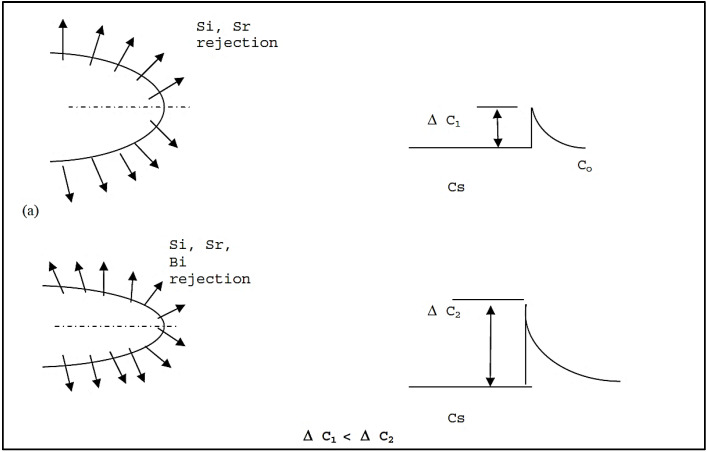

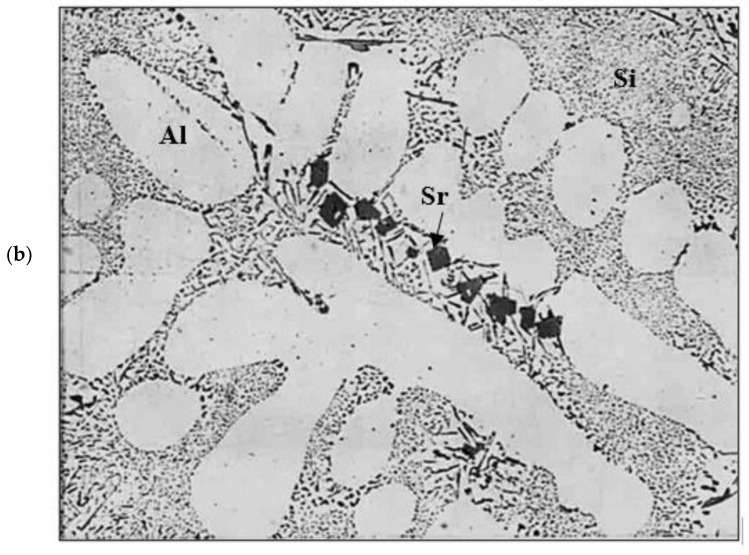
(**a**) Schematic diagram showing rejection of solute atoms in front of a growing α-Al dendrite in B (Sr-modified 319) alloy and Bi-containing B alloy. (**b**) An example of the rejection of Sr and Si in front of developed Al network.

Figure 7a is an attempt to explain this observation, where the growth of an α-Al dendrite in the (Sr-modified) and (Sr-modified + Bi) alloys is shown schematically. In the first case, Si and Sr atoms are rejected in front of the growing dendrite. Consequently, there is more room for the dendrite to grow to a large size, compared to the case below, where the addition of Bi introduces further solute rejection into the surrounding liquid, i.e., ∆C2 > ∆C1. This is seen clearly in Figure 7b. The same explanation can be considered for the case of Ca addition.

The effect of Bi and Ca additions on the interface velocity can be further understood by referring to the Al-Bi and Al-Ca phase diagrams, where the concentrations of both Bi and Ca in aluminum are almost negligible at room temperature. Thus, no solid solution will be formed and all the Bi and Ca will be rejected to the liquid phase. It has been shown that Bi and Ca additions have some effects on the thermal parameters during solidification of Al–Si–Cu alloys: they decrease the thermal gradient (G) and the solidus velocity (R). Important solidification variables, such as average solidification rate (CR) and local solidification time (t_f_) are functions of G and R. The relation between these variables can be expressed as [18]:

CR = G·R = (T_l_ − T_s_)/T_f_ (7)

The secondary dendrite arm spacing (*λ*_2_) varies with the solidification rate according to:

*λ*_2_ = *c* (G·R)^−n^ (8)

*λ*_2_ ∝ CR or freezing time (9)

where *c* and *n* are constants. In general, the exponent *n* lies in the range 0.33 to 0.5 [25,26] This equation allows the solidification rate to be estimated from secondary dendrite arm spacing measurements, which is a direct function of solidification time. Many works on porosity in Al alloys deal with the effect of CR and T_s_ on the process of pore formation and the volume fraction and distribution of pores. It has been reported that the porosity volume fraction for the same hydrogen level increases with increasing local solidification time and decreasing solidification rate. Fraction porosity as calculated from density measurements is found to be inversely proportional to the thermal gradient in the casting and roughly follows a linear relationship with local solidification time. On the other hand, the percent porosity decreases as the feeding efficiency is increased, the latter being defined as [19]:

Feeding efficiency = (G·t_f_^2/3^)/R (10)

The study carried out by Doran [27] shows that the rate of nucleation of new crystals in a supersaturated solution can be expressed by the following empirical equation

B = K_N_ (C − C*) b (11)

where B is the birth rate of new crystals (B represents the number of nuclei formed per unit time and has dimensions T^−1^), *K*_N_ is the nucleation rate constant, C is the concentration of dissolved solute, C* is the solubility of solute at the solution temperature, and b is the order of nucleation. K_N_ and b depend on the physical properties of the system, including the temperature, impurities, and operating conditions. As inferred from Figure 3, molten aluminum has a high affinity to react with O_2_ due to the presence of three electrons in the M shell. Thus, to minimize the oxidation rate of Al during melting, a covering flux composed of equal quantities of KCl and NaCl (melting point is about 658 °C) is used [28]. Prior to pouring, the surface of the molten bath was thoroughly skimmed and quickly poured into the preheated metallic mold (which is placed near the melting furnace) using a preheated metallic ladle (coated with boron nitride) with minimum turbulence.

**Figure 8:**
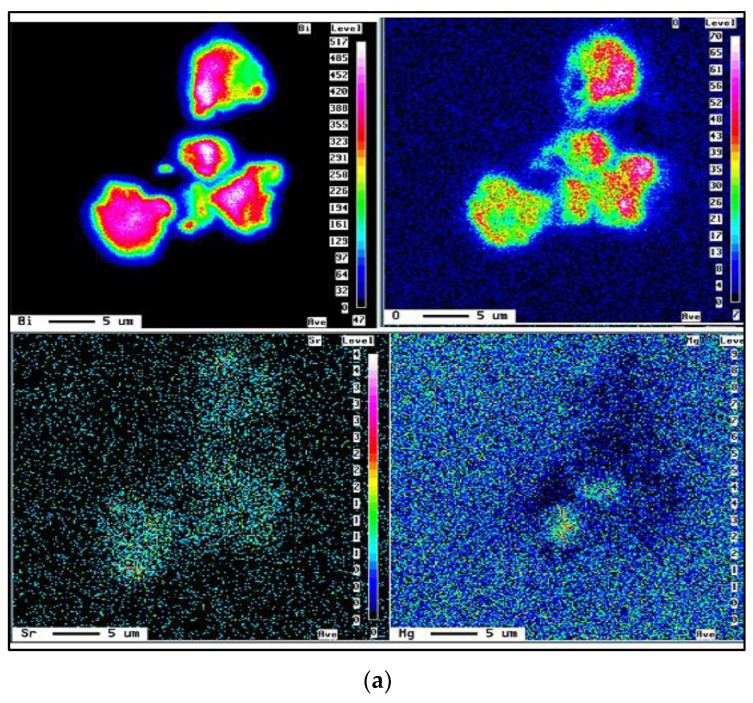

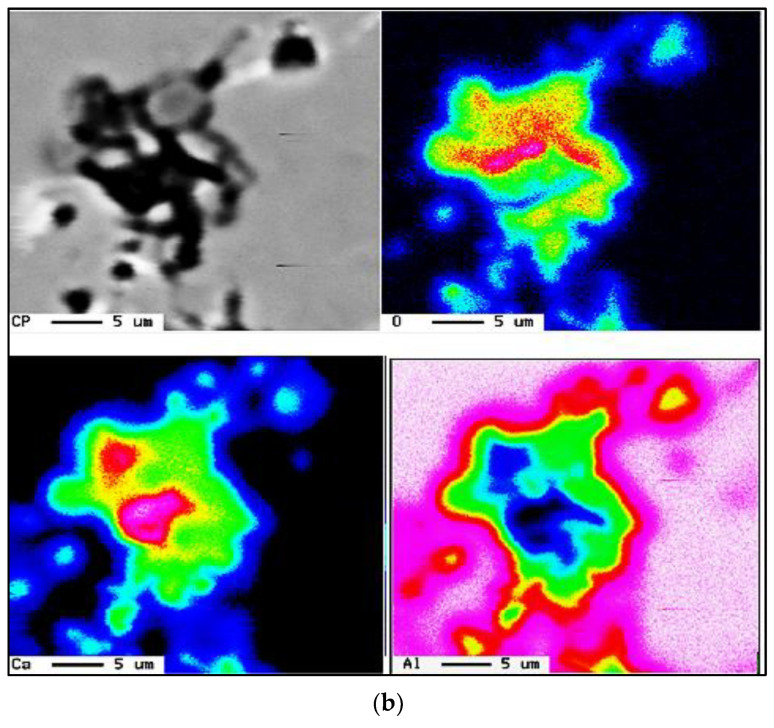
Examples of (**a**) Bi–Sr–Mg–O interactions, (**b**) X-ray mapping of Al-Ca-O particles in the captured picture (CP) shown in (**b**)—see Figure 5f.

The present study attempted to find the reason behind the phenomenon of increased gas porosity in Al cast alloys containing traces of Bi and Ca. The change of thermal parameters, G and R, associated with the addition of Bi and Ca—which results in an increase in local solidification time and a decrease in solidification rate—promotes the formation of gas porosity and is likely one of the reasons for the observed increase in fraction porosity in Al alloys. Other reasons are also recognized: additions of Bi and Ca reduce the surface tension of Al, and thus increase the hydrogen solubility in aluminum. In fact, Bi is one of the most effective elements that reduce the surface tension of Al. The Bi and Ca additions were also found to increase the inclusion content in the metal by their interaction with Mg, Sr, and Al, as shown in Figure 8. These inclusions also act as potential nucleation sites for microporosity formation in the present alloys.

Results on the effect of Bi and Ca additions on the volume fraction and nucleation of pores in the Sr-modified 319 alloys were published by El-Hadad et al. [19]. Their effect on the volume fraction of pores was slight, since the hydrogen content of the melt after 20 min of argon rotary degassing was low. However, many Bi and Ca oxide particles were observed in the solidified structures. The presence of microporosity associated with these oxide particles led us to conclude that the oxide particles acted as preferential sites for pore nucleation. The size of the micropores, however, was small, since additional growth was precluded due to the low hydrogen content. Consequently, the Bi and Ca additions and their effect on solidification parameters were found to have only a minor effect on the porosity volume fraction. Similar observations are found in the literature, where it has been reported that the effect of impurities and inclusions on the volume fraction of pores is only observable at high hydrogen contents of the melt, 0.3 mL/100 g or more. At low hydrogen contents, the inclusion level is of minor importance in porosity formation. On account of the low hydrogen content of the alloy melts in the present case, the influence of the Bi and Ca additions on the solidification parameters was not correlated to the porosity levels. Thus, with respect to gas porosity, hydrogen control is more important than keeping low levels of trace elements [20].

**Figure 9:**
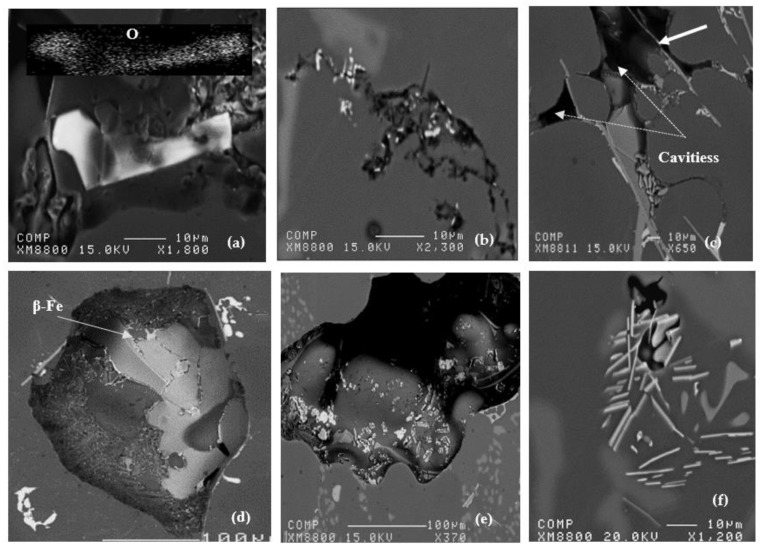
Examples of porosity formed in samples obtained from non-degassed melts related to (**a**) Al_2_SrO_3_ platelet, (**b**) oxide films, (**c**) intermetallics-white arrow, (**d**) gas porosity, (**e**) shrinkage porosity, (**f**) microporosity (large 15°−11.5 cm casting samples).

**Figure 10:**
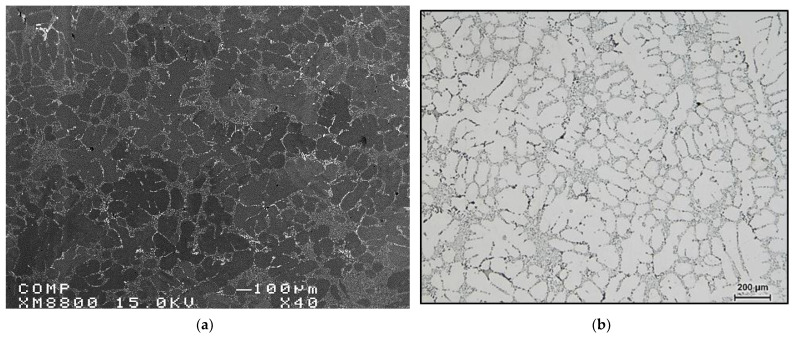
(**a**) Backscattered electron micrograph and (**b**) optical micrograph of a sample obtained from a well-degassed melt.

The origin of pores was attributed to the heterogeneous nucleation on irregular crevices of the oxide particles. It has also been suggested [14,20,23,24] that the origin of pores can be attributed to the growth of the opening of oxide bifilms (double oxide films) by gas precipitating out of solution during solidification. The growth or the opening of bifilms requires almost zero driving force and increases their potential to act as sites for pore initiation. However, in the present case, this is more likely not the reason for the increased porosity observed with the Bi and Ca additions, given the fact that oxide bifilms are found in all cast aluminum alloys and have not been reported to be more extensive in Bi- and Ca-containing alloys. In addition, irregular crevices of oxide particles would have an effect similar to that of oxide films in facilitating the initiation of pores. Regardless of whether any one mechanism is responsible for originating the pores, the influence of Ca and Bi additions on lowering the thermal gradient in the mushy zone and lowering the solidus velocity is still believed to play a dominant role with respect to the increased porosity in aluminum alloys containing traces of Bi and Ca. Figure 9a–f show examples of pores caused by oxides or intermetallics, whereas Figure 10 depicts the microstructure of a sample obtained from a well-degassed melt (clean metal).

### 3.2. Solidification Curves

**Figure 11:**
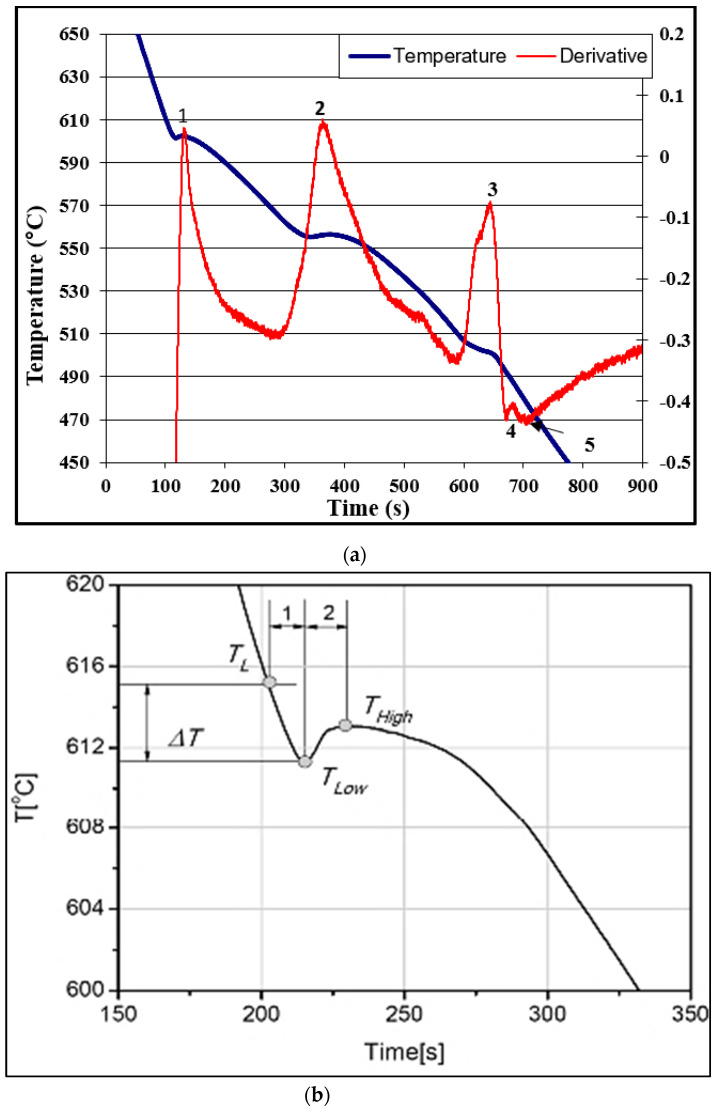
(**a**) Solidification curve and its first derivative (∆T/∆t) of C alloy (0.8 °C/s). (**b**) Schematic diagram of a solidification curve during the beginning of solidification: T_L_: liquidius temperature, T_low_: lowest temperature before recalescence, T_high_: highest temperature after recalescence, ∆Tmaximum undercooling.

Figure 11a depicts the solidification curve of C alloy solidified at a slow rate, approximately 0.8 °C/s, while Table 4 lists the expected reactions [29]. According to Gowri and Samuel [26], the maximum undercooling ∆T is defined as the difference between the liquidus T_L_ and the lowest temperature point T_low_ before the recalescence takes place (∆T = T_L_ − T_low_), see Figure 11b. The maximum undercooling is influenced by the solidification rate.

**Figure 12:**
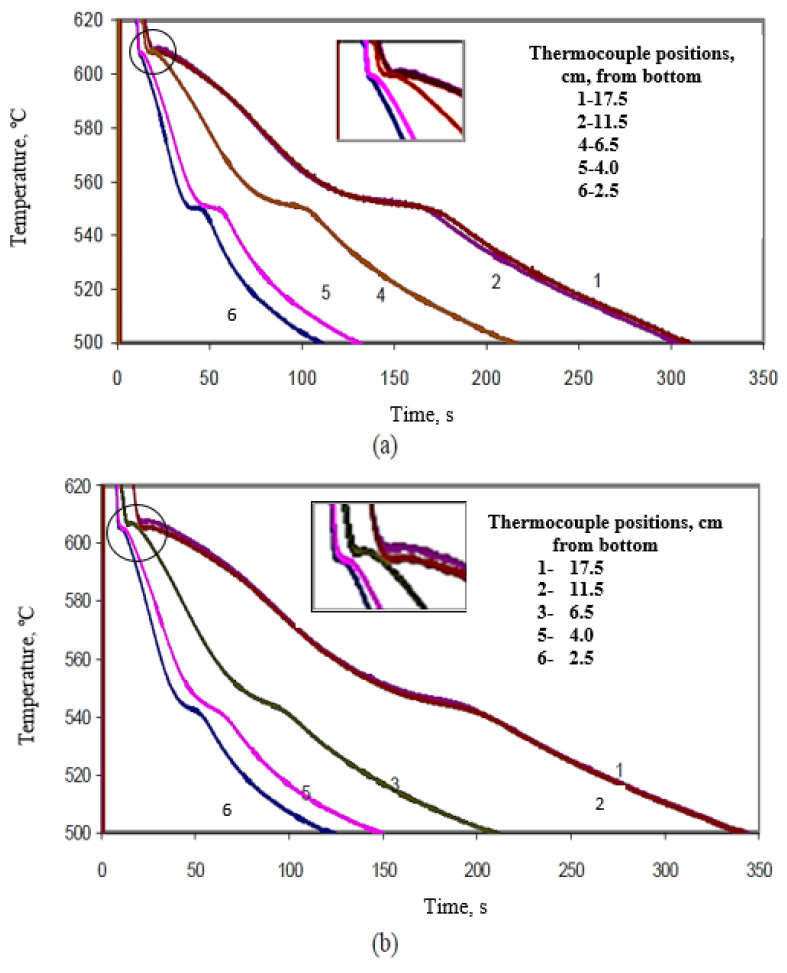
Solidification curves obtained for (**a**) B alloy, (**b**) C alloy, large mold (angle 15°).

**Figure 13:**
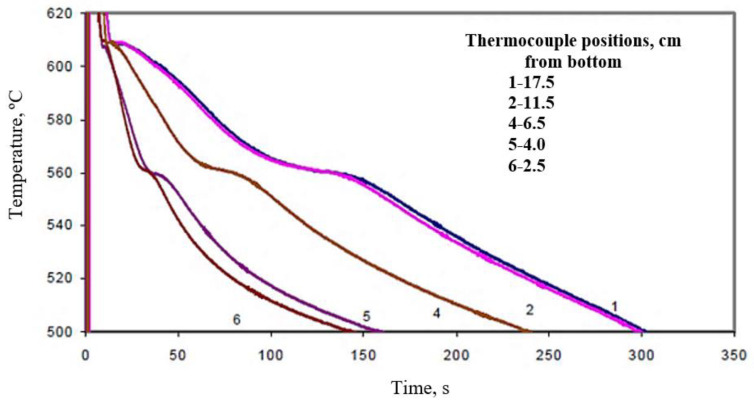
Solidification curves obtained for BB2 alloy (B alloy +2186 ppm Bi, large mold).

Figure 12 shows the temperature-time solidification curves obtained for the large mold (mold angle 15°) for (a) B (Sr-modified), and (b) C (Mg-added B) alloys at the different thermocouple positions, denoted 1 through 6. The effect of Bi addition on the Al-Si eutectic temperature is clearly evidenced in the remarkable increase in the eutectic temperature in the Sr-modified alloy at about 2000 ppm Bi addition, as shown in Figure 13, compared with that observed in Figure 12a for the B alloy. The effect of Bi is less noticeable in the presence of the Mg-added or C alloys. It seems also that the solidification rate has no effect on the Bi-related de-modification action. The Bi addition neutralizes the Sr modification at both slow and fast solidification rates—obtained using different mold angles in this work. Thus, the negative effect of Bi impurity on Si modification can be reliably attributed to the increase in the eutectic temperature associated with the Bi addition.

**Figure 14:**
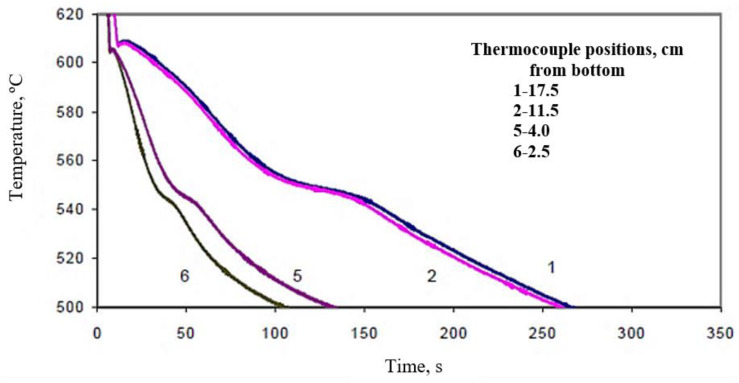
Solidification curves obtained for CC2 alloy (C alloy +94 ppm Ca, large mold).

An example of the effect of Ca addition on the Al-Si eutectic temperature of C alloy is shown by the solidification curves of Figure 14 for CC2 alloy (C alloy + 94 ppm Ca). Comparing Figure 13 with Figure 14 for C alloy reveals that Ca does not noticeably affect the eutectic temperature of the C alloy. No noticeable effect on the eutectic temperature was observed when Ca was added to the B alloy as well. It is important to note here that the solidification curves presented in Figure 12, Figure 13 and Figure 14 were obtained under the same conditions.

Table 5 summarizes the solidification time as a function of alloy composition (large angle mold). Based on Table 5, increasing Mg in A319.2, and hence increasing the number of reactions during solidification (precipitation of Q-Al_5_Cu_2_Mg_8_Si_6_), results in increasing the solidification time. The addition of 94 ppm Ca is more effective in reducing the solidification than the addition of 2000 ppm Bi. This observation may be interpreted in terms of the Ca–Mg interaction in alloy C, as described by Samuel et al. [4]. Since the Mg content in alloy B is about 0.05%, the Bi–Mg interaction is negligible.

### 3.3. Thermal Gradient

**Figure 15:**
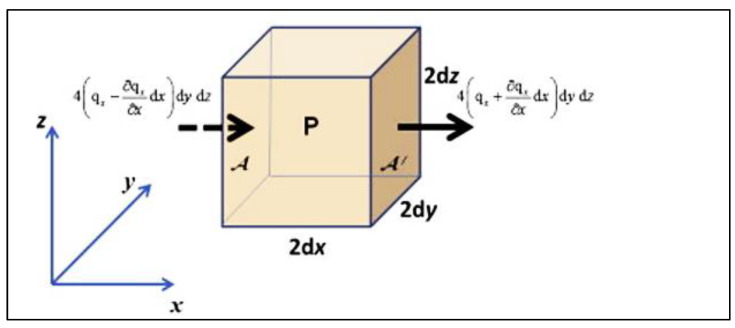
Heat flow in the x-direction through an element of volume at P.

Temperature gradient is inversely proportional to the thermal conductivity [29]. In mathematical form [10],

δT∝−k

where δT is the temperature gradient and *k* is the thermal conductivity. The negative sign shows that the temperature decreases. Therefore, the heat transfers in the positive direction. A positive sign indicates that the temperature increases, so that the heat transfers in the negative direction. Thus, if the temperature gradient is positive, heat transfers in the negative direction and vice versa. An example is shown in Figure 15.

**Figure 16:**
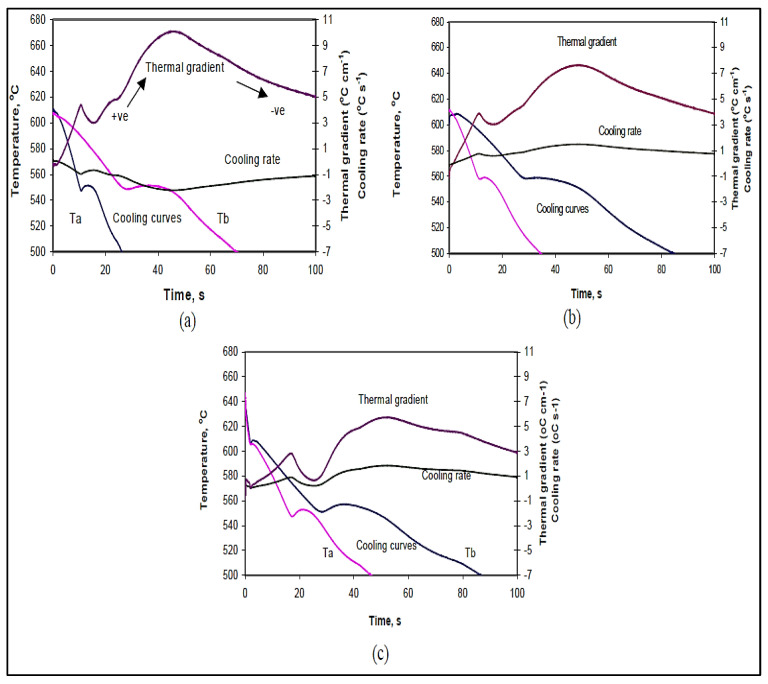
Comparison of temperature, thermal gradient, and solidification rates versus time for (**a**) B (Sr-modified 319), (**b**) BB2 (B + 2186 ppm Bi), and (**c**) BC2 (B + 103 ppm Ca) alloys (small mold).

The average vertical thermal gradients (G), as a function of the distance from the bottom of the mold (6.5 cm), were plotted for the different alloy compositions. The simultaneous comparison of temperature, local temperature gradient, and solidification rate, using graphs of the type shown in Figure 16, facilitated the recognition of specific temperatures. As described previously, a positive thermal gradient corresponded to the case when the uppermost thermocouple (T_b_) of the six thermocouples used registered a higher temperature than the lowest one (T_a_), with respect to the bottom of the mold. It should be mentioned here that the temperature coded T_a_ was measured using a second thermocouple to avoid hot zones.

During solidification, the change in thermal gradient is almost positive for the alloy conditions studied, as seen from Figure 16. The figure also shows that the thermal gradients increase with the solid fraction in the mushy zone. Thus, the thermal gradient is minimum in the liquid state and maximum at the end of solidification. In the solid state, the change in thermal gradient decreases with time, indicating that thermal equilibrium is being approached.

In addition, it is clear from Figure 16 that the addition of Bi and Ca to the Sr-modified 319 alloy lowers the thermal gradient during solidification and subsequent cooling. The effect of Ca is found to be more remarkable than that of Bi (cf. Figure 16b,c). A lowering of the thermal gradient will take place if the addition widens the size of the mushy zone (keeping the solidus and liquidus temperatures unchanged). At the same solidification conditions, the size of the mushy zone can also increase when the liquidus temperature decreases. The liquidus temperature is the temperature of the Si eutectic in the present alloys.

The additions of Bi and Ca at levels corresponding to those shown in Figure 16 were seen to increase the eutectic temperature of the base alloy. Therefore, the lowering of the liquidus temperature is not the reason for the observed decrease in the thermal gradient during solidification. It can then be deduced that Bi and Ca alter the thermal properties of the solid or liquid, most likely by decreasing the thermal conductivity of either one or the other. Both Bi and Ca have partition coefficients less than unity in aluminum (they are less soluble in the solid phase than in the liquid phase) [30,31]. The enrichment of the liquid with Bi and Ca during solidification and the observed reduction in thermal gradient in the mushy zone suggests that these elements likely decrease the thermal conductivity of liquid aluminum, which is the continuous phase in the mushy zone, leading to an increase in the mushy zone size and a subsequent decrease in the thermal gradient.

Nevertheless, the observed decrease in the thermal gradient during solidification and the expected increase in the mushy zone size do have an effect on the porosity formation in these alloys [32]. The volume fraction of microporosity, which has been previously reported to grow in a diffusion-controlled manner, should increase in alloys with large mushy zones. Thus, the addition of Bi and Ca, which lower the thermal gradient during solidification, give more time for the extremely fast hydrogen diffusion in liquid aluminum to take place, and, therefore, promote the formation of a high volume fraction of microporosity. In addition, it has been previously reported that low solidification rates and small thermal gradients give rise to porosity formation in Al–Si and Al–Si–Cu alloys. The observations of increased porosity in Bi and Ca-containing Al alloys can, therefore, be attributed in part to the influence of these additions on lowering the thermal gradients during solidification.

### 3.4. Interface Velocity

The velocity of the solid interface could be determined through knowledge of the time at which the thermocouple in a location reached the solidus temperature. The time t_2_ it took for the thermocouple immediately above to reach this temperature was also recorded. The distance between the two thermocouples divided by the (t_2_ − t_1_) time interval gave the interface velocity in cm/s. An average solidus velocity was calculated for each sample in the same way the average temperature gradient was calculated.

**Figure 17:**
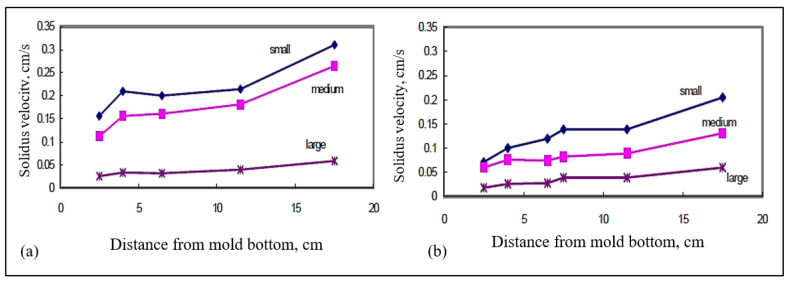
Average vertical solidus interface velocities in the wedge mold as a function of the distance from the bottom of the mold for (**a**) B alloy, (**b**) BB2 alloy (B + 2186 ppm Bi) for small, medium, and large molds.

**Figure 18:**
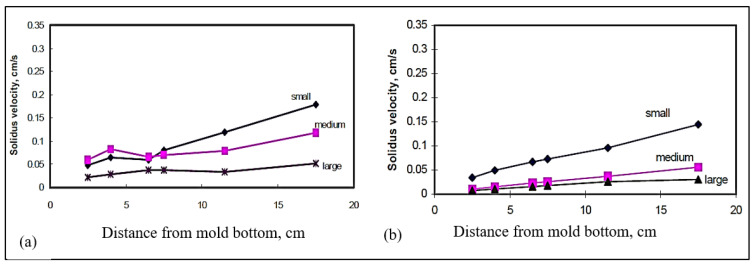
Average vertical solidus interface velocities in the wedge mold as a function of the distance from the bottom of the mold for (**a**) C alloy, (**b**) CB6 alloy (C + 3020 ppm Bi) for small, medium, and large molds.

**Figure 19:**
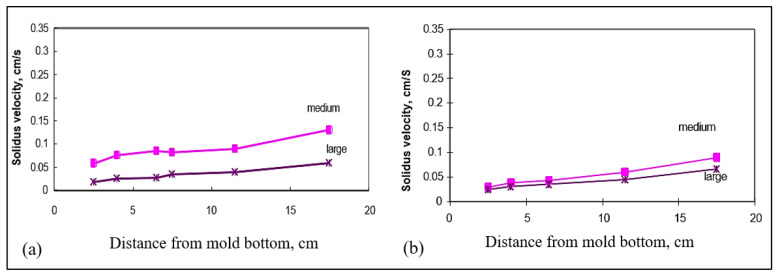
Average vertical solidus interface velocities in the wedge mold as a function of the distance from the bottom of the mold for (**a**) BC2 alloy (B + 103 ppm Ca), and (**b**) CC2 alloy (C + 94 ppm Ca), for medium and large molds.

The average vertical solidus velocity (R) was also measured for the different castings. Figure 17a,b illustrate the average vertical solidus interface velocities obtained as a function of the distance from the bottom of the mold for (a) B (Sr-modified 319), and (b) BB2 (B + 2186 ppm Bi) alloys, respectively. The velocities were measured in the small, medium, and large molds. From these two figures, we can observe that the interface velocities are reduced with the addition of Bi to the B alloy. Similar observations were found in the case of the C alloy, Figure 18. Calcium affects the interface velocity in a similar fashion, as can be seen from Figure 19, and compared with Figure 17a and Figure 18a. Due to difficulties associated with the measurements in the small mold configuration (i.e., thermocouple failures), only the plots for the medium and large molds are shown in Figure 19.

## 4. Conclusions

The effects of Bi and Ca as impurities in Sr-modified A319.2 and B319.2 casting alloys on the solidification thermal parameters were investigated in this work. Based on the results obtained, the following conclusions could be drawn.

1. The addition of Bi increases the Si-eutectic temperature, while no noticeable effect on the eutectic temperature is observed when Ca is added, indicating that Bi has a higher affinity to react with Sr than does Ca.
2. The additions of Bi and Ca lower the thermal gradient in the mushy zone during solidification. In addition, Bi and Ca lower the solidus velocities.
3. The solidus interface velocity is affected by the rejection of solute atoms in front of the moving solid/liquid interface. This observation is more obvious in the small mold (0° angle) section than in the large mold (15° angle) section, due to the much higher velocity obtained in the former case.
4. In the absence of oxides, the effect of Bi and Ca additions on the solidification thermal parameters constitutes an important reason for the observed changes in Si morphology and porosity volume fraction.
5. Both BiO and CaO form very fine microporosity (less than 1µm). Their fine size and also the concentration of such microporosity in/near the hot spots, particularly in the case of the small mold castings, explains the low percentage porosity values obtained in the B and C alloys with Bi and Ca additions.

## Figures and Tables

**Table 1:** Chemical compositions (wt.%) of the two main alloys used in the present work.

Alloy	Si	Cu	Fe	Mg	Mn	Ti	Zn	Sr	Al
B *	6.14	3.63	0.11	0.048	<0.0005	0.14	<0.0017	0.0152	Bal
C **	6.16	3.48	0.10	0.635	<0.0015	0.15	<0.0017	0.0118	Bal

* A319.2 alloy, ** B319.2 alloy.

**Table 2:** Alloy codes for Bi- and Ca-containing alloys.

Alloy Code	Alloy Used + Addition	Alloy Code	Alloy Used + Addition
BB	B + 0621 ppm Bi	CB6	C + 3020 ppm Bi
BB2	B + 2186 ppm Bi	BC	B + 0052 ppm Ca
BB4	B + 3785 ppm Bi	BC2	B + 0103 ppm Ca
BB6	B + 4060 ppm Bi	BC5	B + 0465 ppm Ca
CB	C + 0464 ppm Bi	CC	C + 0063 ppm Ca
CB1	C + 0874 ppm Bi	CC2	C + 0094 ppm Ca
CB2	C + 1034 ppm Bi	CC5	C + 0486 ppm Ca
CB4	C + 2264 ppm Bi		

**Table 3:** (a) Porosity characteristics of Sr-modified alloys—variable angle mold (small). (b) Porosity characteristics of Sr-modified alloys—variable angle mold (15° large).

(a)								
Alloy Code	Area Percentage Porosity		Pore Characteristics					
			Area (µm^2^)		Length (µm)		Density (#pores/mm^2^)	
	Average	S. D.	Average	S. D.	Average	S. D.		
BB	0.065	0.047	152.5	58.6	08.5	11.6	162	
BB2	0.078	0.016	119.4	29.3	13.5	13.6	152	
BB4	0.092	0.054	506.1	87.4	33.9	31.3	179	
BB6	0.062	0.025	320.4	33.3	32.1	25.9	75	
CB	0.129	0.245	929.8	321.3	60.3	38.2	240	
CB1	0.231	0.220	833.0	371.9	48.7	40.3	275	
CB2	0.210	0.151	927.1	83.2	48.1	42.7	222	
CB4	0.192	0.128	590.6	29.8	39.6	24.6	155	
BC	0.086	0.084	455.8	67.1	28.0	22.7	184	
BC2	0.008	0.008	104.7	24.4	11.7	12.1	81	
BC5	0.177	0.178	655.7	44.3	47.4	31.2	163	
CC5	0.258	0.223	562.9	27.6	38.3	25.9	410	
**(b)**								
**Alloy** **Code**	**Pore Characteristics**							
	**Area** **(µm^2^)**			**Length** **(µm)**			**Density** **(#pores/mm^2^)**	
	**Average**		**S. D.**	**Average**		**S. D.**		
BB	160.2		82.0	17.8		09.3	155	
BB2	174.7		28.4	22.5		13.2	468	
BB4	123.1		69.6	18.8		10.2	652	
BB6	184.3		25.7	22.5		10.2	575	
CB	244.6		35.0	25.6		18.8	237	
CB1	343.0		78.1	18.4		16.4	749	
CB2	243.2		78.9	14.7		8.1	822	
CB4	287.0		53.6	35.0		19.5	474	
BC	182.8		88.5	12.4		11.2	142	
BC2	153.1		57.7	15.6		17.1	276	
BC5	146.2		63.1	22.2		12.4	550	
CC5	266.7		67.8	16.4		11.7	478	

Note: Mold temperature: 325 °C; Small angle: 0°, Large angle: 15°; S. D.: Standard deviation.

**Table 4:** Expected phases in C alloy solidified at 0.8 °C/s.

Reaction #	Temperature (°C)	Type of Reaction
1	601	Precipitation of α-Al network
2	561.2	Al-Si eutectic reaction
3	504.2	Al-Al_2_Cu eutectic reaction
4	498.1	Precipitation of Al_5_Mg_8_Cu_2_Si_6_
5	483	End of solidification

**Table 5:** Solidification time as a function of alloy composition and thermocouple position (large angle mold).

Figure #	Alloy Composition	Thermocouple Position (cm)	Solidification Time (s)
12a	B	17.5	300
		11.5	300
		6.5	210
		4.0	120
		2.5	100
12b	C	17.5	330
		11.5	330
		6.5	200
		4.0	130
		2.5	115
13	BB2	17.5	280
		11.5	330
		6.5	200
		4.0	130
		2.5	115
14	CC2	17.5	250
		11.5	250
		4.0	110
		2.5	90

## Data Availability

Data will be made available upon request.
